# The Technique of a Modified Ponte Osteotomy Using Ultrasonic Bone Scalpel in Spinal Deformity Correction: Does It Save Time and Reduce Blood Loss?

**DOI:** 10.7759/cureus.77858

**Published:** 2025-01-22

**Authors:** Ajay Krishnan, Vikrant Chauhan, Sandesh Agarawal, Bharat Dave, Degulmadi Devanand, Mirant B Dave, Shivanand Mayi, Ravi R Rai, Abhijith Anil, Mikeson Panthackel, Kishore Murkute

**Affiliations:** 1 Spine Surgery, Stavya Spine Hospital and Research Institute, Ahmedabad, IND; 2 Spine Surgery, Bhavnagar Institute of Medical Science, Bhavnagar, IND; 3 Orthopaedics, Government Doon Medical College and Hospital, Dehradun, IND; 4 Orthopaedic Surgery, Sri Devaraj Urs Academy of Higher Education and Research, Kolar, IND; 5 Orthopaedics/Spine Surgery, Stavya Spine Hospital and Research Institute, Ahmedabad, IND

**Keywords:** blood loss, deformity, osteotomy, ponte, spine, ultrasonic bone scalpel

## Abstract

Background and objective

Posterior column osteotomies, such as Smith-Petersen and Ponte osteotomies, are widely utilized in the surgical correction of spinal deformities to address sagittal and coronal imbalances by releasing the posterior tension band. While traditional methods using rongeurs and osteotomies are effective, these are often associated with prolonged operative time, significant blood loss, and increased risk to neural structures. These challenges have driven interest in advanced tools like the ultrasonic bone scalpel (UBS), which uses high-frequency vibrations to enable precise bone cutting with minimal damage to surrounding tissues. This innovative tool has demonstrated significant reductions in blood loss and operative time in various spinal procedures. However, its specific application in modified Ponte osteotomies remains underexplored. This study evaluates the UBS's safety, efficiency, and impact on blood loss in complex deformity corrections and presents a refined technique for optimizing outcomes in these challenging surgeries. Additionally, it outlines a refined technique for executing modified Ponte osteotomies with this advanced tool.

Methods

This retrospective study included all patients who underwent spinal deformity surgery involving modified Ponte osteotomy with a UBS between January 2013 and June 2022. Key metrics analyzed included the number of modified Ponte osteotomy segments performed per surgery, the total time taken for osteotomies, and the average time required per segment. Blood loss was evaluated with a focus on epidural and bony sources, and methods for controlling bleeding were documented. Safety parameters included intraoperative neurophysiological signal integrity and the incidence of dural tears.

Results

A total of 695 modified Ponte osteotomy segments were performed in 111 patients undergoing complex spinal deformity correction surgery. The average number of segments created per procedure was 6.26 ± 1.59 (range: three to nine). The mean operative time required for all segments per surgery was 19.16 ± 5.66 minutes, with an average time of 3.05 ± 0.29 minutes per segment (range: 2.13-4.66 minutes). Epidural bleeding was observed at an average of 1.65 ± 1.27 points per surgery (range: 0-6), equating to 0.26 ± 0.20 points per segment. Bleeding was effectively controlled with bipolar ablation and absorbable gelatin sponge packing, and no significant bony bleeding was reported. Of note, there were no instances of intraoperative neuromonitoring signal loss or dural tears, demonstrating the safety of the technique. No splinters occurred while osteotomizing in any case.

Conclusions

The use of UBS significantly enhances the efficiency and safety of modified Ponte osteotomy during spinal deformity correction surgeries. This technique not only reduces operative time but also minimizes blood loss, offering an advanced approach to achieving precise osteotomies without compromising patient safety. These findings underscore the benefits of incorporating UBS into complex spinal surgical procedures.

## Introduction

Posterior column osteotomy techniques, such as Smith-Petersen osteotomy (SPO) and Ponte osteotomy, have been widely utilized for correcting spinal deformities involving sagittal and coronal imbalance thanks to their ability to release the posterior tension band [1,2]. Ponte osteotomy has gained popularity in various spinal deformity correction procedures, including adolescent idiopathic scoliosis, owing to its efficacy in restoring coronal and sagittal balance [3-5]. However, performing modified Ponte osteotomy using traditional instruments like Leksell or Kerrison rongeurs and osteotomes is often associated with prolonged operative time and increased estimated blood loss [4-6]. The ultrasonic bone scalpel (UBS), a tool increasingly used in spine surgery, has been well-documented for procedures such as laminectomy, laminoplasty, facetectomy, and corpectomy. It offers enhanced precision and facilitates complex deformity corrections and ossifying disorders while maintaining utility in routine procedures [7-15]. UBS has been observed to reduce overall blood loss, provide a wider safety margin to minimize the risk of dural tears and neural deficits, and improve operative efficiency [13-16].

Reports of the use of UBS-assisted modified Ponte osteotomy have focused primarily on its impact on estimated blood loss. Bartley et al. reported a statistically significant reduction in estimated blood loss - by 30-40% - compared to traditional osteotomes for Ponte osteotomy [17]. However, Garg et al. found no significant reduction in blood loss during inferior facetectomies performed with the UBS compared to traditional methods [18]. The purpose of this retrospective study was to primarily evaluate the safety, estimated blood loss, and quickness of performing modified Ponte osteotomy as a part of its use in spinal deformity correction surgeries. The secondary objective was to describe an improvised technique of modified Ponte osteotomy with a UBS.

## Materials and methods

Study design

This study was a retrospective observational analysis of patients who underwent modified Ponte osteotomy for deformity correction surgery from January 2013 to June 2022 at our institution with the use of a UBS. This study is exempted from the institutional review board approval due to its retrospective observational nature.

Inclusion and exclusion criteria

The inclusion criterion was as follows: patients aged 9-21 years with spinal deformity who underwent modified Ponte osteotomy as part of deformity correction surgery for scoliosis using UBS. The Exclusion criteria were as follows: (1) adult degenerative scoliosis; (2) patients with coagulopathy; (3) revision surgery; (4) kyphosis or ankylosed spine where sagittal correction and/or ventral decompression was the primary goal; (5) scoliosis where modified Ponte osteotomy was not done; and (6) patients aged less than nine years.

Surgical technique

All the procedures were performed at the same hospital by a single surgeon (A.K). The patient was positioned prone on the standard radiolucent operative table on the chest and pelvic roll under general anesthesia and intraoperative neuromonitoring involving somatosensory evoked potential, transcranial motor evoked potential, free running electromyography, and pedicle screw testing (NIM Eclipse, Medtronic, Memphis, TN). A standard midline posterior incision was used and subperiosteal tissue dissection was performed to expose the spinous process, lamina, articular process, and transverse processes. Pre-planned posterior instrumentation and pedicle screw insertion were completed by both freehand technique and under an intraoperative CT-based navigation system (Stealth S8 Navigation System, Medtronic Sofamor Danek, Memphis, TN).

Modified Ponte osteotomy at each level was performed in a sequential stepwise manner using a UBS (Misonix, Farmingdale, New York, NY), osteotomes rongeurs under optical loupes magnification and illumination [HEINE Optotechnik, Gilching, Germany (2.5x magnification, 520 mm focal length, 55,000 lux illumination)]. For cutting bone with UBS, incomplete bony cuts were initially made. The bony cut was then completed by introducing a thin corticotome through the incomplete bony cut (corticotomy) and twisting it gently, thereby breaking the remaining inner cortex (osteotomy) [10]. For modified Ponte osteotomy, firstly, bilateral inferior facetectomy was performed in the thoracic or lumbar spine via two perpendicular cuts (one transverse cut along pars and one longitudinal cut along lamino-facet junction using UBS).

**Figure 1:**
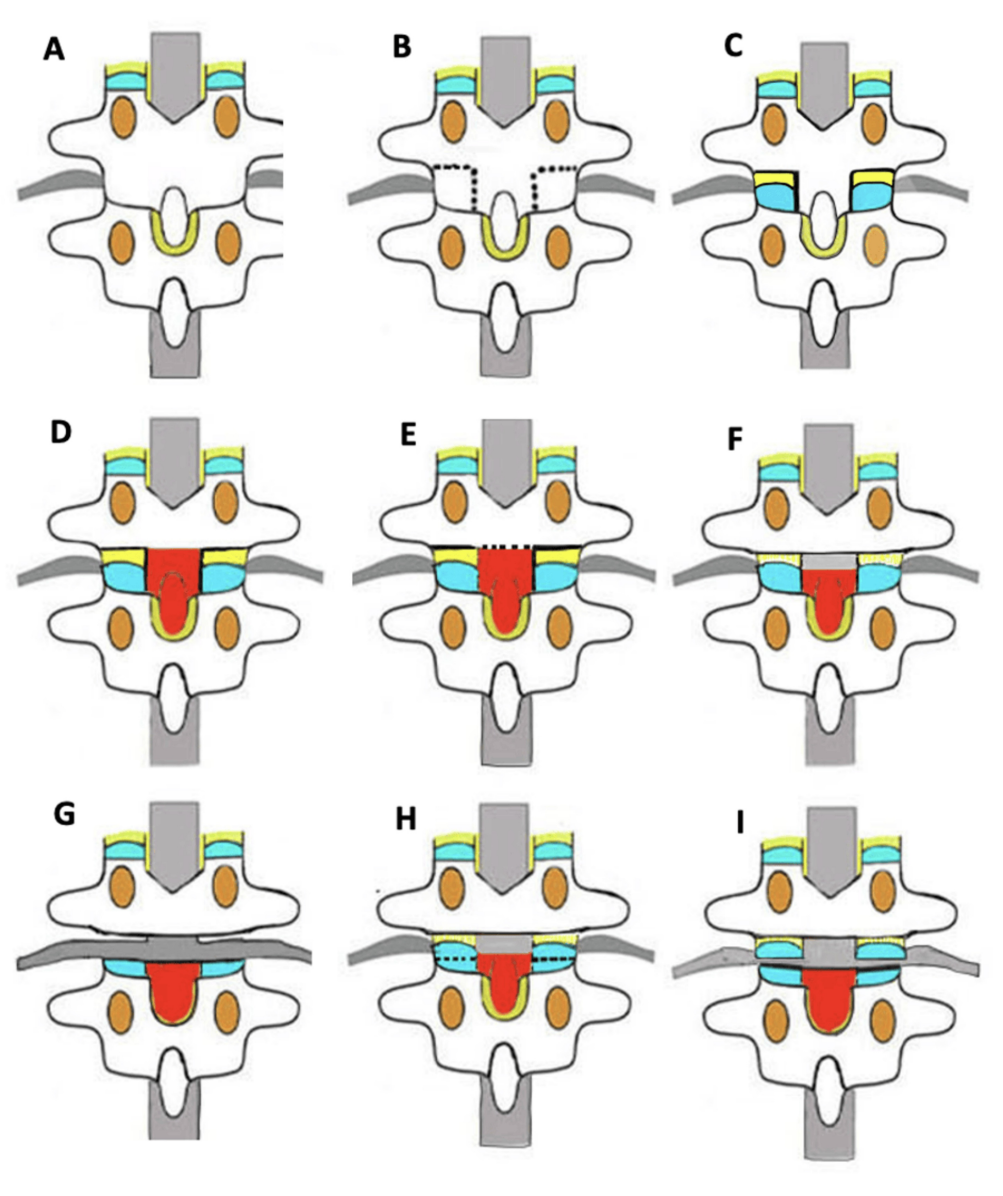
Illustrations showing the steps of UBS-assisted modified Ponte osteotomy (A) Pictorial representation of the intact vertebrae. (B) Inferior facet corticotomy cuts with UBS are done bilaterally (dotted line). (C) Inferior facet osteotomy is complete & the facet bone section is removed with Leksell's rongeur. The blue color represents the visualized articular cartilage of the superior articular process. The lateral ligamentum flavum & capsular tissue of the facet (yellow) are visible. (D) The spinous process and lamina nibbled with the rongeur. The red color is the raw bone surface. (E) A horizontal corticotomy cut (dotted line) is made in the central lamina, with UBS. (F) The laminar osteotomy is complete, & the free segment of the raw spinous process and lamina are displaced down due to the elastic tension of intact ligamentum flavum below. The spinal cord gets exposed (gray). The lateral ligamentum flavum & capsular tissue of the facet are the only connecting structures posteriorly. (G) The facet & lateral ligamentum tissues were removed with Kerrison's rongeur. Bleeding is expected at this stage, as the footplate of the rongeur enters into the neural canal. The spinal cord with the root gets exposed (gray) after excision. Now, Ponte osteotomy is complete. After 2022, the author has further modified the procedure (H and I). We have started cutting the superior articular process also horizontally (dotted) with UBS. So, in image H, it can be noted that the corticotomy is done. In image I, the corticotomy is completed & the half segment of the superior articular process and ligamentum flavum remain separated & in situ rather than getting excised with rongeur as described till image G. This reduces the bleeding further as the rongeur usage is curtailed (Dotted line): corticotomy cuts. The bone is cut with the ultrasonic scalpel but not detached. (Complete line: the incomplete corticotomies connected by twisting the corticotome. The osteotomy is complete and the bone section is separated UBS: ultrasonic bone scalpel Image credit: The authors

The expected free segment was decorticated using Leksell rongeur before the osteotomy cuts as it can be difficult to decorticate a free rattler later. A transverse cut was then made using UBS for detachment of the lower two-third part lamina and spinous process connecting to inferior facetectomy cuts on either side, thus releasing the superior attachment of ligament flavum from adjoining lamina and creating a rattler. Central ligamentum flavum is non-existent at this osteotomy site. The fragment with ligament flavum was kept in situ to avoid exposure of the thecal sac in cases where excessive corrections of deformity were not needed. Otherwise, for severe deformity, it was excised. Superior articular facet osteotomy was performed with UBS and, with the ligamentum flavum/capsule, it is excised using the rongeur. The epidural bleeding points were controlled using an absorbable gelatin sponge and/or bipolar ablation when noted. Bone bleeding points were controlled with bone wax or ablation. The steps of modified Ponte osteotomy using UBS are illustrated in detail in Figures 1A-1I.

Rod persuasion and reduction maneuvers included various combinations of differential rod contouring, cantilever, apical vertebral derotation, compression/distraction, and in situ bending to achieve the planned optimum target correction. Decortication of the rest of the lamina, pars, and facet region was done with burr/and rongeurs. Morselized bone grafting was done using local autograft bone. Closure was performed in layers under a negative suction drain with interrupted and running vicryl in the deep fascia and deep dermal layer and Monocryl for the subcuticular layer.

Variables assessed

Demographic details and surgical variables such as patient age, gender, and surgical diagnosis were extracted and analyzed. The number of modified Ponte osteotomy segments performed in a patient was noted with the total time taken to perform the whole modified Ponte osteotomy. Then per-segment time was also calculated. Bleeding was noted by the total number of epidural bleeding points observed requiring bipolar ablation and/or gelatin sponge packing and/or bony bleeding points management with bone wax during the modified Ponte osteotomy. Safety was noted with the assessment of signal loss in intraoperative neuromonitoring before and after the completion of modified Ponte osteotomy. Dural tears, if any, or thermal blackening of dura, or any occurrence of splinter while osteotomizing were noted.

Statistical analysis

All statistical analyses were performed using SPSS Statistics version 25.0 (IBM Corp., Armonk, NY). Means ± standard deviation (range) was calculated for all continuous variables. Statistical significance was set at p<0.05. An unpaired t-test was employed to compare continuous variables with a normal distribution. Pearson's correlation coefficient was calculated to identify if any relation existed between the number of modified Ponte osteotomy segments created and the average epidural bleeding segment requiring intervention.

## Results

**Figure 2:**
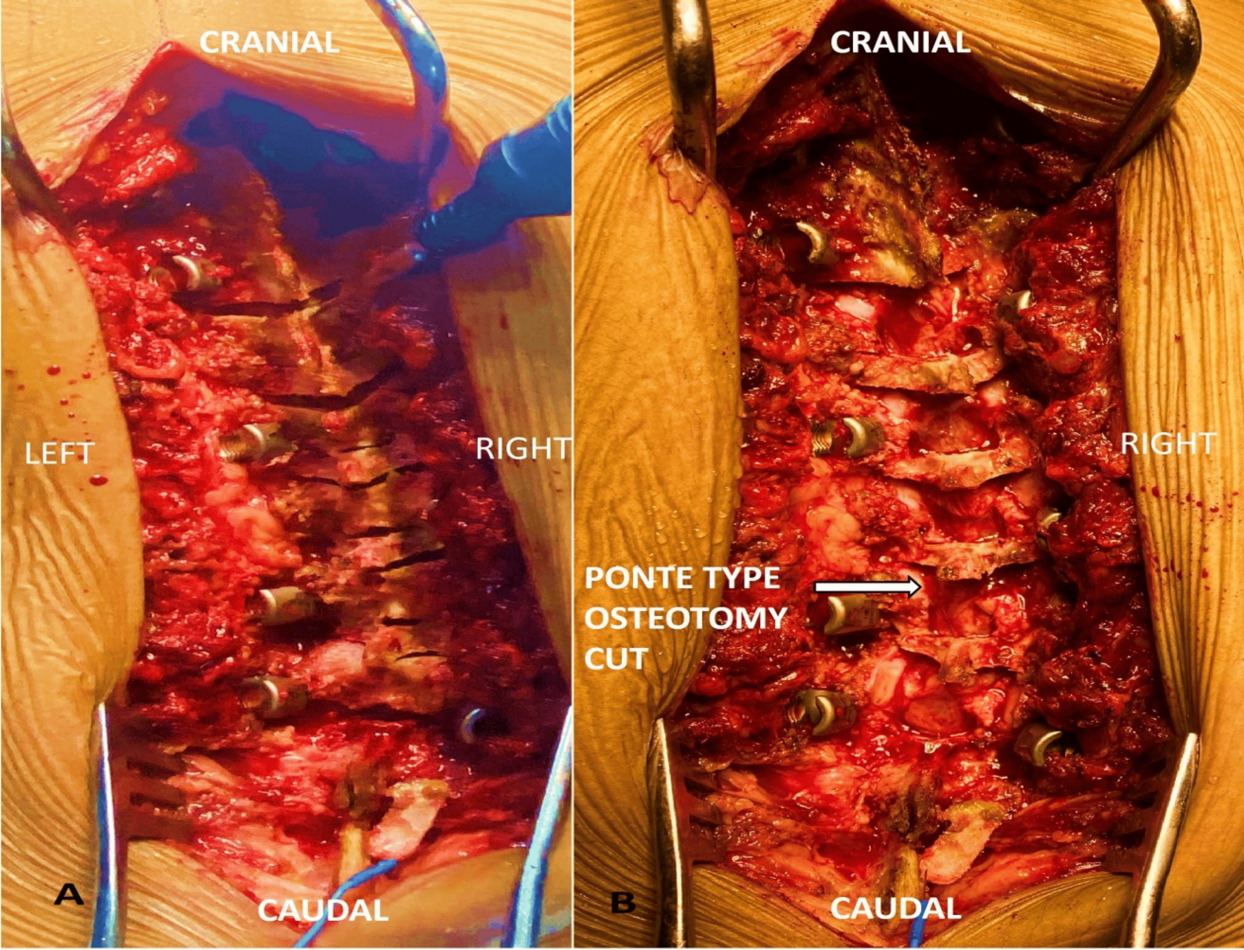
Surgical images (A) Transverse cut of modified Ponte osteotomy made in lamina made using UBS. (B) Post en-block excision of the laminar intersegmental bone and facet UBS: ultrasonic bone scalpel

Per inclusion and exclusion criteria, 111 patients underwent modified Ponte osteotomy using UBS for spinal deformity correction surgeries from January 2013 to June 2022 as exclusive or in addition to another major osteotomy. The mean age of the patients was 14.79 ± 2.21 (9-21) years. Of note, 82 (73.87%) patients were female and 29 (26.12%) were males. The most common spinal deformity for which modified Ponte osteotomy was done for correction was idiopathic scoliosis (n=80) followed by neuromuscular scoliosis (n=13), as shown in Table 1. The demographic variables of the cohort are presented in Table 2. A total of 695 modified Ponte osteotomy segments were created using UBS as illustrated in Figure 2.

**Table 1:** Pathology findings (n=111)

Pathology	N (%)
Congenital scoliosis	5 (4.50%)
Idiopathic scoliosis	80 (72.07%)
Neurofibromatosis	4 (3.60%)
Neuromuscular	13 (11.71%)
Syndromic	4 (4.50%)
Kyphosis	2 (1.80%)
Kyphoscoliosis	3 (2.70%)

**Table 2:** Demographic characteristics

Variables	Values
Male/female	29/82
Average age, years	14.79 ± 0.04
Average no. of Ponte segments	6.26 ± 1.59
Total time/case, minutes	19.16 ± 1.35
Average time/segment, minutes	3.05 ± 0.29
Average epidural bleeding points	1.65 ± 2.72
Average epidural bleeding point/segment	0.27 ± 0.07

The number of modified Ponte osteotomy segments created per surgery was 6.26 ± 1.59 (range: three to nine) and the time to perform a modified Ponte osteotomy segment per surgery was 19.16 ± 5.66 (range: 11-35) minutes. The time to perform each modified Ponte osteotomy segment was 3.05 ± 0.29 (range: 2.13-4.66) minutes. The epidural bleeding points noticed per surgery was 1.65 ± 1.27 (range: 0-6) and the epidural bleeding point per modified Ponte osteotomy segment created was 0.26 ± 0.20 (0-0.85), which required bipolar ablation and absorbable gelatin sponge packing to control bleeding. No bony bleeders were noted. A higher number of modified Ponte osteotomy segments created per surgery was correlated with a significant increase in average epidural bleeding points requiring intervention (Pearson’s correlation coefficient: 0.44; p=0.041. Regarding the safety of UBS in modified Ponte osteotomy creation, no dural tear was noted and there were no signal losses in intraoperative neuromonitoring observed during osteotomy.

## Discussion

SPO and Ponte osteotomy are both widely used techniques for correcting spinal deformities, but differences in their terminology and procedural specifics often lead to confusion. In the Schwab osteotomy classification system, SPO is categorized as a grade 1 osteotomy. Originally introduced in 1945 to treat flat lumbar spine deformities in ankylosing spondylitis, SPO is an opening wedge osteotomy. It involves anterior column osteoclasis and posterior column shortening through the removal of facet joints, with the middle column serving as a pivot. On the other hand, Ponte osteotomy is classified as a grade 2 osteotomy and is primarily employed to correct sagittal plane deformities, such as those seen in Scheuermann’s kyphosis. This technique involves a wide resection of facet joints as well as of laminae and a complete removal of the ligamentum flavum [19-21]. Unlike SPO, which relies on anterior column osteoclasis during osteotomy closure, Ponte osteotomy necessitates a mobile anterior column for effective correction. These distinctions in technique and biomechanical principles emphasize the importance of choosing the appropriate osteotomy based on the underlying deformity and surgical goals [22]. In this study, we altered the traditional approach to Ponte osteotomy.

Although Ponte osteotomy was initially introduced for correcting primary hyperkyphosis, its application at multiple levels in scoliotic deformities has proven to enhance spinal flexibility. This increased flexibility facilitates improved correction of both coronal and sagittal plane deformities [23]. The advantages of Ponte osteotomy in managing scoliotic deformities are well-documented in the literature [24]. For instance, Feng et al. compared the outcomes of multiple-level Ponte osteotomy with posterior soft tissue release in idiopathic scoliosis and found that the Cincinnati correction index was significantly better in the Ponte osteotomy group. Similarly, Shah et al. demonstrated that Ponte osteotomy significantly enhances coronal and sagittal parameters during corrective surgery for adolescent idiopathic scoliosis.

Increased intraoperative estimated blood loss during deformity correction surgeries is influenced by multiple factors, including male gender, preoperative hemoglobin levels, preoperative Cobb angle, longer operative time, the number of anchor points, and the inclusion of additional corrective osteotomies [25-27]. While effective, Ponte osteotomy is conventionally associated with higher intraoperative estimated blood loss. Feng et al. compared Ponte osteotomy with soft tissue release in idiopathic scoliosis surgeries and reported a statistically significant increase in intraoperative estimated blood loss in the Ponte osteotomy group. Similarly, Halanski et al. observed higher intraoperative estimated blood loss and longer operative time in the Ponte osteotomy group compared to the group undergoing only inferior facetectomies during deformity correction surgeries.

In the literature, conventional instruments such as osteotomes and rongeurs are commonly used to create Ponte osteotomy segments during deformity correction surgeries. However, unlike UBS, these tools do not seal or coagulate the marrow interstices, resulting in greater intraoperative oozing. Additionally, rongeurs and osteotomes remove a larger volume of bone, along with attached soft tissue, which can lead to the avulsion of epidural veins and increased blood loss [28]. Bartley et al. reported that using UBS for facetectomies and apical Ponte osteotomy, compared to standard osteotomes and rongeurs, reduced overall blood loss by 30 to 40% [17]. Similarly, Wahlquist et al. found that UBS use decreased estimated blood loss by 26% in idiopathic scoliosis cases and 45% in neuromuscular scoliosis cases [29].

In our study, only one out of every four Ponte osteotomy segments created required intervention, such as bipolar ablation or gelatin sponge packing, to control epidural bleeding. No bony bleeding was observed in any of the cases, and the bleeding noted was significantly less than that associated with conventional Ponte osteotomy methods. The use of UBS has been described in the field of oral and maxillofacial surgeries for precision cuts and avoidance of injury to adjacent neural and vascular structures [30,31]. Selective cutting of rigid tissue like bone and avoidance of entrapment of adjacent soft tissue (a common occurrence with motorized high-speed burr) led to the expansion of indication of UBS in other surgical branches. In the field of spine, the use of UBS for the removal of bone (facetectomies, corrective osteotomies) has been successfully demonstrated and described in the literature. In a study by Onen et al., which involved a comparative analysis of the use of UBS and high-speed burr in cervical laminectomies, the UBS group had lesser intraoperative blood loss and operative duration, and the difference was statistically significant.

UBS operates by converting electrical energy into mechanical energy through piezoelectric transducers. This process produces a back-and-forth motion of the blade at a frequency of 22,500 Hz, effectively pulverizing and cutting mineralized tissues like bone [32]. A key advantage of UBS is its tissue selectivity, owing to the differential mineralization of tissues. While it efficiently cuts bone, soft tissues like the ligamentum flavum and dura absorb the vibrations, minimizing the risk of injury. This feature is particularly beneficial in the cervical and thoracic regions, where traditional instruments like Kerrison rongeurs may cause mechanical spinal cord injury or avulsion of epidural veins due to repeated canal entry. UBS avoids these issues by removing bone in a single piece [33]. Also, the heat generated during bone cutting with UBS coagulates the marrow interstices, sealing bleeding edges and significantly reducing blood loss and surgical morbidity [14,15]. UBS employs a narrow blade with a self-irrigating system that provides lubrication and cooling within the cutting cavity, further reducing mechanical and thermal injury risks [34]. In our study, the time taken per modified Ponte osteotomy segment was 3.05 ± 0.29 minutes, with minimal interventions needed to stop bleeding points (0.27 ± 0.07 per segment). These findings support the efficiency of UBS in achieving rapid segmental osteotomy with significantly reduced blood loss.

UBS assists additionally well in blood loss reduction by avoiding injury to the epidural veins by allowing a more precise technique. Other adult deformity corrections may also immensely benefit from the use of UBS and may negate the need for blood product transfusion, which is in alignment with enhanced recovery after surgery protocols of spine surgery [35]. Three further modifications, which are not part of the current study, have also now routinely been used at our institute since 2022: differential complete facet cutting on the convex side for asymmetric gain of the osteotomy and not removing the superior facet and lateral ligamentum flavum/capsule attached with the osteotomised facet to reduce further epidural bleeding points which can come while excising it with the rongeur. This has further reduced the time per segment (Figure 1H-1I).

While UBS is an advanced surgical tool, its ergonomic design and intuitive operation may enable relatively quick adoption, even by surgeons accustomed to conventional instruments. However, studies evaluating the learning curve and its effect on operative efficiency are necessary to refine training protocols [36]. Future research should focus on examining the learning curve associated with UBS and its impact on surgical performance over time. Additionally, long-term outcomes of UBS-assisted osteotomies, including effects on spinal alignment, fusion rates, and patient-reported quality of life, should be further investigated. Randomized controlled trials comparing UBS with other osteotomy techniques are crucial for definitively determining its role in reducing perioperative complications and enhancing surgical outcomes. Moreover, the use of UBS in multi-level osteotomies for scoliotic deformities, especially in correcting coronal and rotational imbalances, requires further exploration.

Limitations

Our study has several limitations. Precise quantification of estimated blood loss was not possible due to the water-based nature of UBS, which makes it challenging to measure fluid and blood loss accurately, as this would also include continuous oozing from the exposed soft tissues and bone surfaces during extensive surgical exposure. Therefore, estimated blood loss was not directly measured. Instead, we relied on subjective methods, such as observing bleeding points at the epidural and visualizing bleeding at the cut bone ends, to assess blood loss. This contrasts with previous studies, which employed more objective methodologies for total blood loss measurement [4,6,17,18,29]. However, those studies considered overall surgical blood loss, not just the Ponte osteotomy segment. Additionally, coagulants like Floseal, which could further reduce blood loss, were not used in our study as they are not part of the protocol at our institute. The contribution of UBS to coronal, sagittal, and rotational deformity correction was not assessed in this study, as it was not the primary focus. Furthermore, the study involved 111 cases, and variations in estimated blood loss due to patient heterogeneity were not analyzed because of small subgroups. Despite these limitations, UBS can still be considered a valuable tool in the deformity surgeon’s armamentarium, as it helps reduce blood loss and operative time significantly. Indirectly, it may also contribute to reduced perioperative morbidity and costs.

## Conclusions

UBS is a valuable tool for performing modified Ponte osteotomy safely and efficiently. It reduces blood loss and maintains precision without compromising safety. Indirectly, it reduces surgical time. Hence, UBS should be considered an essential addition to the spine surgeon's armamentarium for deformity correction surgeries. Further research is needed to validate its long-term benefits and optimize its use.
